# A method for reproducible measurements of serum BDNF: comparison of the performance of six commercial assays

**DOI:** 10.1038/srep17989

**Published:** 2015-12-10

**Authors:** Alessio Polacchini, Giuliana Metelli, Ruggiero Francavilla, Gabriele Baj, Marina Florean, Luca Giovanni Mascaretti, Enrico Tongiorgi

**Affiliations:** 1 Department of Life Sciences, University of Trieste, 34127 Trieste, Italy; 2 Department of Transfusion Medicine, Trieste University Hospital, 34142 Trieste, Italy

## Abstract

Brain-Derived Neurotrophic Factor (BDNF) has attracted increasing interest as potential biomarker to support the diagnosis or monitor the efficacy of therapies in brain disorders. Circulating BDNF can be measured in serum, plasma or whole blood. However, the use of BDNF as biomarker is limited by the poor reproducibility of results, likely due to the variety of methods used for sample collection and BDNF analysis. To overcome these limitations, using sera from 40 healthy adults, we compared the performance of five ELISA kits (Aviscera-Bioscience, Biosensis, Millipore-ChemiKine^TM^, Promega-Emax^®^, R&D-System-Quantikine^®^) and one multiplexing assay (Millipore-Milliplex^®^). All kits showed 100% sample recovery and comparable range. However, they exhibited very different inter-assay variations from 5% to 20%. Inter-assay variations were higher than those declared by the manufacturers with only one exception which also had the best overall performance. Dot-blot analysis revealed that two kits selectively recognize mature BDNF, while the others reacted with both pro-BDNF and mature BDNF. In conclusion, we identified two assays to obtain reliable measurements of human serum BDNF, suitable for future clinical applications.

The availability of biomarkers to support the diagnosis or monitor the efficacy of therapies is a major unmet clinical need in neurology and neuropsychiatry^1^. Indeed, in spite of the large number of published studies on the association between brain disorders and molecular markers present in biological fluids, only a few clinically useful biomarkers have been successfully validated for the routine clinical practice^2^,^3^,^4^. The neurotrophin Brain-Derived Neurotrophic Factor (BDNF) is one of the most promising biomarkers for brain disorders however, a definitive clinical validation is still lacking. BDNF is a secretory, dimeric growth factor present in most human tissues, including brain and blood^5^. BDNF is known to play a fundamental role in survival and differentiation of selected neuronal populations during development, and in the maintenance and plasticity of neuronal networks during adulthood^6^,^7^. Similar to other neurotrophins, BDNF is first synthesized as a precursor protein, named pro-BDNF of 32 KDa, which is cleaved by different proteases to produce either the mature form of 14 KDa, or the truncated form of 28 KDa. Interestingly, an altered balance of the different forms has been linked with cognitive impairment and psychiatric disorders^8^,^9^,^10^.

Meta-analyses and reviews of clinical studies based on the measurement of BDNF in whole blood, serum, or plasma have reported significantly lower BDNF levels in patients with major depression^11^,^12^, schizophrenia^13^, bipolar disorders^14^, or autism spectrum disorders^15^,^16^. These reviews however, highlighted severe discrepancies among studies, which even reported opposed results (increase vs. decrease or no change). Serum or plasma BDNF levels are increased by antidepressant treatments^11^,^17^,^18^,^19^. In addition, recent exploratory studies have found increased serum BDNF levels following holistic neuro-rehabilitative approaches, including computer-assisted cognitive enhancement in schizophrenia^20^, aerobic exercise in stroke^21^ and mindfulness clinical trials in bipolar-disorder^22^. Interestingly, these studies showed differences in collecting the samples that may add an additional variability other than the well known variables, such as BMI, drugs, smoking, etc.^23^, affecting circulating BDNF levels, thus resulting in further difficulties in assessing BDNF levels related to the pathology or treatment. It would be then of great advantage to have a shared methodology concerning the pre-analytical stage (sample preparation and storage), the analytical stage (analysis execution) or assay-related (intrinsic assay quality), in order to compare the BDNF levels. Therefore, this study is aimed at providing a comparison of some commercially available assays to measure BDNF in human serum. Accordingly, we measured BDNF concentration in sera from 40 healthy volunteers using 6 different commercial kits and comparing their performances.

## Results

**Figure 1:**
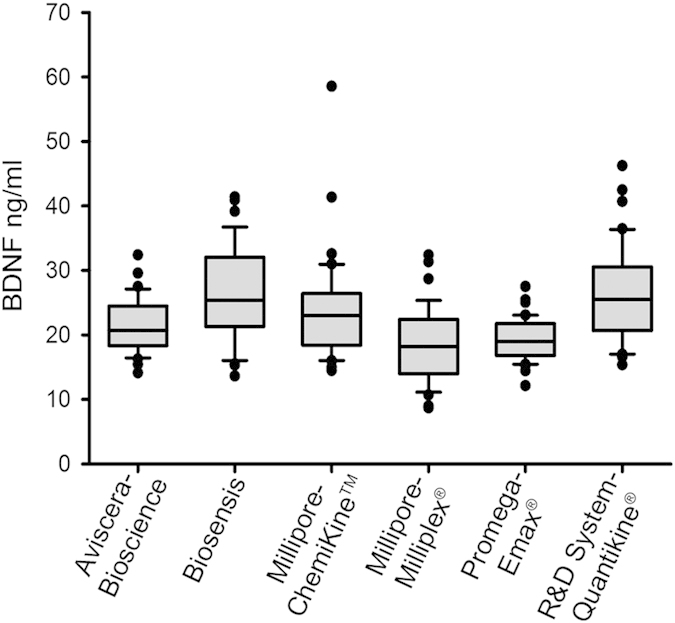
BDNF serum levels measured with the indicated ELISA assays. Box plot of serum BDNF concentrations (ng/ml) from healthy volunteers (n = 38–40, see Table 2) represented as mean of two independent measures. The upper line of the box marks the 75th percentile, the middle line is the median value and the lower line specifies the 25th percentile. Whiskers above and below the box indicate the 90th and 10th percentiles, respectively. Dots indicate the outlier values within each group.

Serum samples were prepared from adult subjects, whose blood was withdrawn after overnight fasting^23^,^24^, between 8:00 and 12:00^25^,^26^,^27^,^28^,^29^, and allowed to clot for 1 h at room temperature and 1 h at 4 °C^30^,^31^. Following centrifugation at 2000 g for 10 min at 4 °C, serum samples were stored at −80 °C^23^,^32^ in aliquots of 50 μl in thin wall 0.2 ml PCR tubes arranged in strips of 8 tubes with attached flat lid (Sarstedt, Multiply^®^ μStripPro). We compared the performance of five sandwich ELISA assays from different companies (Aviscera-Bioscience, Biosensis, Millipore-ChemiKine^TM^, Promega-Emax^®^ and R&D System-Quantikine^®^) and one multiplexing assay (Millipore-Milliplex^®^). The main characteristics of the kits and their performance, as declared by the manufacturers, are described in Table 1. Using the six kits, we assessed BDNF concentration in the sera prepared from 40 healthy blood donors (mean age 54 ± 6 years; 18/22 Females/Males ratio). These measurements were carried out at controlled room temperature (24° ± 1 °C) and repeated measures were carried out by the same experimenter using two aliquots of the same sera stored at −80 °C and then thawed only once at the time of usage. Samples were diluted following the recommendations provided in each kit (see Table 1) and corresponded to 1:40 for Aviscera-Bioscience, 1:200 for Biosensis, Millipore-ChemiKine^TM^ and Promega-Emax^®^, 1:10 for Millipore-Milliplex^®^ and 1:20 for R&D System-Quantikine^®^. Whenever a positive control and/or an additional point of the standard curve were requested by the kit, the samples tested were lowered to 38 (Aviscera-Bioscience and Millipore-Milliplex^®^) or 39 (Biosensis). Sample recovery was 100%, as we always found measurable concentrations of BDNF, irrespectively of the kit used. Distributions of values were not normal and therefore data are shown as box plot with indicated median value and 10^th^, 25^th^, 75^th^, 90^th^ percentile (Fig. 1). Serum BDNF concentrations resulted to be in the same range for all kits (Fig. 1, Table 2). Median values and percentiles of BDNF concentrations were comprised between a minimum of 18.2 (14.2-22-2) and a maximum of 25.5 (21–30.5) ng/ml (Table 2).

**Figure 2:**
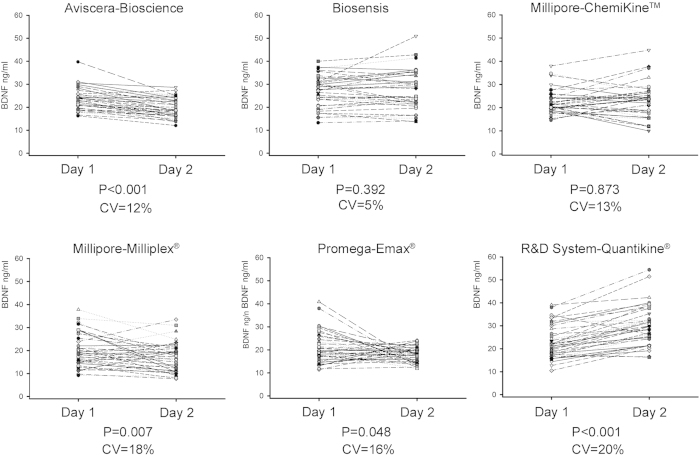
Inter-assay variation of the BDNF ELISA kits. Scatter plot showing the BDNF values distribution measured by the same operator on two different days using two plates of the same lot for each brand (Day 1 & Day 2). Each dot represents a BDNF value from one subject and the dashed lines link together two assessments of the same subject. The reproducibility was checked performing one-way ANOVA for repeated measures and the P values are specified.

Intrinsic assay quality was evaluated by testing the intra-assay and the inter-assay coefficients of variation (CV) for each kit (Table 2). Intra-assay CV was assessed by comparing BDNF values measured twice into the same plate, for each subject. Five kits presented values within the declared CV, with the only exception of Millipore-Milliplex^®^ which showed a higher than expected intra-assay CV (13–14% versus <10%). Measurements of serum BDNF were repeated on a different day by the same experimenter using two different plates of the same lot and then, inter-assay reproducibility was verified by one-way ANOVA for repeated measures (Fig. 2). With the only exception of Biosensis (p = 0.392) and Millipore-ChemiKine^TM^ (p = 0.873), which showed reproducible results, four kits provided significantly different results of serum BDNF assessments between Day 1 and at Day 2 (Fig. 2). The inter-assay CV tested showed values higher than the declared ones, with the exception of Biosensis (5% tested, versus 5–8% declared) (see Table 2). Of note, the Aviscera-Bioscience and Millipore-ChemiKine^TM^ kits showed inter-assay CV values moderately higher than those declared (12% vs. 8–10%, and 13% vs. 9%, respectively) while the inter-assay CVs for Millipore-Milliplex^®^ (16%), Promega-Emax^®^ (18%) and R&D System-Quantikine^®^ (20%) were well above the declared values (Table 2).

**Figure 3:**
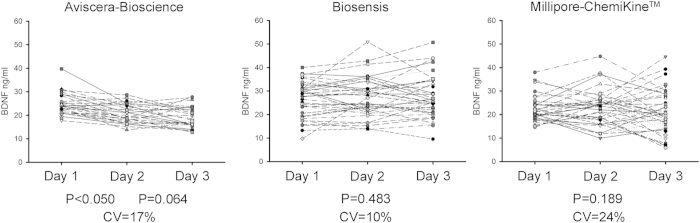
Inter-assay variation of the Aviscera-Bioscience, Biosensis and Millipore-ChemikineTM kits over a third replica. Scatter plots show the BDNF values distribution measured by the same operator on three different days for each brand. Distributions for Day 1 and Day 2 are the same as Fig. 2, here reported for comparison; distributions for Day 3 were obtained 1 year after Day 2, using a plate of a different lot (serum samples were stored at −80 °C). The reproducibility was checked performing one-way ANOVA for repeated measures and *post-hoc* Bonferroni correction applied when a significant comparison was found. P values and cumulative CVs expressed in percentage are given.

Since, Aviscera-Bioscience, Biosensis and Millipore-ChemiKine^TM^ showed the best inter-assay CV values, these kits were further tested for their reproducibility in a third replica which was carried out at 12 month after the second test (Fig. 3). Statistical analysis with one-way ANOVA for repeated measures, followed by a Bonferroni correction, revealed no statistically significant differences for the Biosensis and Millipore-ChemiKine^TM^ kits even after the third replica, while for the Aviscera-Bioscience kit there was no difference only between the second and the third replica (Fig. 3). The cumulative inter-assay CV resulting from the triplicate assessment were 17% for Aviscera-Bioscience, 10% for Biosensis and 24% for Millipore-ChemiKine^TM^. This experiment, also allowed us to establish the perfect conservation state of the samples after one year storage at −80 °C, in agreement with previous studies^23^,^32^ .

These results suggested that the Biosensis kit provides the most reproducible results. However, we decided to submit this kit to one further test combining an analysis of intra-assay CV at different sample dilutions and a test for operator-dependent reproducibility. In this experiment, four serum samples were diluted at 1:50, 1:100, 1:200 and 1:400 and tested by two operators working in parallel, using the same materials. The dilution at 1:50 resulted to give saturation for all samples, for both operators, and therefore these data were excluded from further analysis. Using the 1:100, 1:200 and 1:400 dilutions the overall intra-assay CV was 11% for operator-1 and 8% for operator-2 (p = 0.146; not statistically significant, one way ANOVA for repeated measures). Within this range of dilutions the samples’ O.D. were fitting the linear region of the standard curve.

**Figure 4:**
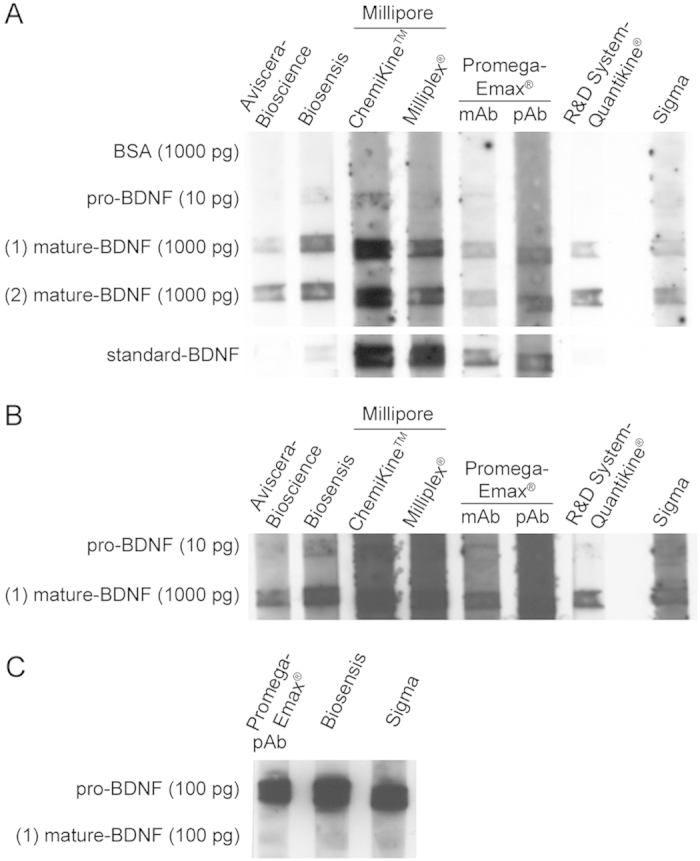
Line-blot for qualitative analysis of anti-BDNF antibodies specificity. (**A**) The antibodies from each ELISA kit were tested for specificity against pro-BDNF or mature BDNF. The BDNF standards blotted were commercial pro-BDNF (Alomone; 10 pg/lane), mature BDNF (1 and 2 from Alomone and Sigma, respectively; both 1000 pg/lane) and the standard BDNF protein included in each kit (Aviscera-Bioscience and Biosensis: 10 pg/lane; Millipore-ChemiKine^TM^, Millipore-Milliplex^®^- and R&D System-Quantikine^®^: 100 pg/lane; Promega-Emax^®^: 1000 pg/lane). BSA (1000 pg/lane) was used as a negative control. The mouse monoclonal anti-BDNF antibody, (1:1000; Sigma) was tested as a control. (**B**) Central region of the same blot shown in A, from an overexposed film to better visualize the reactivity against pro-BDNF. (**C**) Reactivity of antibodies from Biosensis, Promega-Emax^®^ pAb and Sigma on a dot blot in which the same quantity of pro-BNDF and mature BDNF were spotted (100 pg each). Each antibody from the ELISA kits was used at the dilution suggested by the manufacturer’s instructions. mAb: Promega-Emax^®^ monoclonal capture antibody for plate coating. pAb: Promega-Emax^®^ polyclonal detection antibody.

To complete the assessment on the assay performance, we verified if the detection antibodies of each kit were able to recognize mature BDNF or pro-BDNF, or both, in a dot blot assay (Fig. 4). In the case of the Promega-Emax^®^ kit, also the capture monoclonal antibody was available (mAb). As a negative control, we spotted on the same membrane strip Bovine Serum Albumin (BSA) and, as a positive control, the BDNF standard provided in each kit (standard-BDNF; Fig. 4A). In addition, a monoclonal anti-BDNF antibody commercially available from Sigma was tested on purified mature BDNF or pro-BDNF. Results demonstrated similar reactivity of the antibodies against commercial mature BDNF from Alomone Labs or Sigma (1000 pg). Biosensis, Millipore (both kits) and Promega-Emax^®^ antibodies reacted also with purified pro-BDNF while those from Aviscera-Bioscience and R&D System-Quantikine^®^ showed only marginal reactivity to pro-BDNF (Fig. 4A). Of note, Aviscera-Bioscience and R&D System-Quantikine^®^ kits were claimed to be specific for mature BDNF and to have some cross-reactivity with the human pro-BDNF. Since the tested antibodies resulted to be very reactive against pro-BDNF, the spotted quantity of purified pro-BDNF was reduced (1:100) respect to mature BDNF and therefore spotted as 10 picograms. To better evaluate reactivity against pro-BDNF, the central region of the dot-blot shown in Fig. 4A was overexposed (Fig. 4B; 10 pg of pro-BDNF). Moreover, Figure 4C shows the reactivity of Promega-Emax^®^ (pAB), Biosensis and Sigma against the same amount of pro-BDNF and mature-BDNF (100 pg) spotted on the substrate, highlighting a stronger reactivity of these antibodies against pro-BDNF with respect to mature-BDNF.

**Figure 5:**
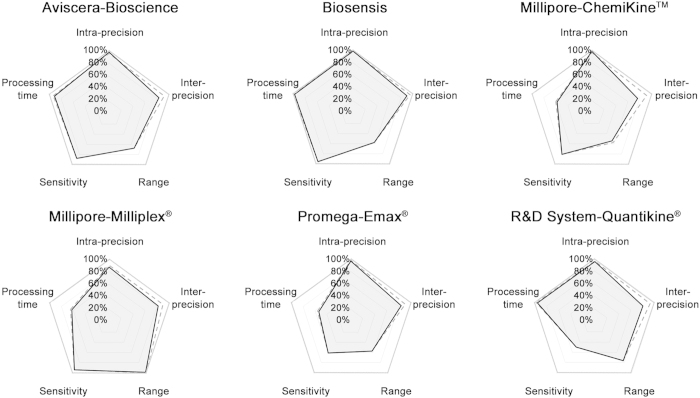
Graphic summary of BDNF ELISA kit performances. Polar plot showing the kit performances. The black solid line indicates the assessed performances, the dashed line shows those declared by the manufacturers, while bold grey line indicates the best values (100%). Data are from a triplicate experiment for Aviscera-Bioscience, Biosensis and Millipore-ChemiKine^TM^ while from a duplicate for the other kits. See text for a detailed description.

To compare the six BDNF kits examined, we drew polar plots showing the kit performance for intra-precision, inter-precision, range, sensitivity and processing time (Fig. 5). The black solid line indicates the assessed performances, while the dashed line shows those declared by the manufacturers. The best value for each factor was set as the reference (100%; bold grey line). The value for intra-precision was set as 100% subtracted by the measured intra-assay CV (e.g. for Aviscera-Bioscience: 100%-2% = 98%). The value for inter-precision was set as 100% subtracted by the measured inter-assay CV (in analogy to the intra-assay CV). The range was defined as the logarithm of the “highest detectable value – lowest detectable value” declared by the manufacturer and divided by the logarithm of the largest range which was set as 100%. Since in Millipore-ChemiKine^TM^ and Millipore-Milliplex^®^ kits, the most concentrated standard reduced the linearity of the curve of about 6% and 2% respectively, the range values of these two kits were corrected accordingly. The kit Millipore-Milliplex^®^ has the largest range (Table 1). The values for sensitivity were stated equal to the declared one, since all kits displayed an absorbance of at least 10% above background. Moreover, to obtain a graphical comparison, we defined a theoretical best detection sensitivity as 1 pg/ml (set as 100%) and a theoretical worst one as 40 pg/ml (set as 0%). Then, the values for sensitivity were calculated as follows: sensitivity = 100 − (value in pgml/40 pgml * 100); e.g. for Aviscera = 100 − 12.5 = 87.5%). The processing time values were normalized against the best declared value set as t = 3 hours (100%) over an arbitrary worst value of 48 hours (0%).

## Discussion

In the present study we propose a highly reproducible BDNF quantification method potentially suitable for clinical applications. To obtain a reduction in the variability of measurements due to technical issues, we identified the best performing assay for BDNF detection by comparing six widely used commercial kits based on the ELISA technique, the most common way to measure circulating BDNF and, according to our results, one of the main sources of inconsistencies among studies.

A recurrent question among researchers is whether it is more correct to measure BDNF levels in plasma, serum or whole blood. For this study we decided to measure circulating BDNF in the serum. There are several reasons that support this choice. First, BDNF concentration in serum is about 100 folds higher than plasma levels^33^. Second, BDNF concentration in plasma is affected by handling of the blood sample because of the presence of platelets, which store BDNF and upon degranulation can secrete it^34^,^35^. Simple shearing forces produced by the needle during blood withdrawal can cause platelet degranulation and even changes in room temperature and timing can produce significant release of BDNF in the plasmatic fraction^30^. In addition, it has been shown that because of the release from platelets, BDNF concentration raises progressively within few hours from plasma preparation^30^,^33^. Consequently, the BDNF quantification from plasma can be sensitive to preparation procedures and is very difficult to be reproducible among different operators. Secondly, release of BDNF from platelets can be influenced by age, specific disease conditions, or pharmacological treatments, which may be difficult to control^35^,^36^. Concerning the whole blood, although measurement of BDNF in serum and whole blood give comparable results, we agree with Elfing and colleagues^30^ that, since whole blood must be lysed before the BDNF measurements, the cell lysis step may add additional variability during sample preparation. In conclusion, since BDNF is not produced in megakaryocyte precursor cells but actively picked up by platelets from the circulating BDNF pool^34^,^37^,^38^, it can be assumed that serum may reflect the totality of circulating BDNF. Indeed, serum BDNF concentration is almost identical to the amount of BDNF in washed plateled lysates^34^,^36^. In addition, while BDNF circulate in plasma for less than an hour, platelets circulate as much as 11 days^39^,^40^.

The performance for each kit in term of intra-assay variation, inter-assay variation, detection range, sensitivity and processing time are graphically summarized in Fig. 5. Among the six kits, the Biosensis and Aviscera-Bioscience, which detect total BDNF and mature BDNF respectively, were the most performant and displayed the largest area closer to the 100% of values for the five parameters (Fig. 5). In particular, Biosensis, showed the lowest coefficient of variation even after the third replica (10%) and no significant difference between three independent measurements. Of note, the Promega-Emax^®^ kit was previously demonstrated to give inter-assay CV values which were ranging from 8.5%^41^, in line with declared values by manufacturer, to more than 24%^42^ highlighting a strong experimenter-dependence for the performance of this kit, which is the only requiring the manual coating of the plate. In terms of CV, Millipore-ChemiKine^TM^ and Aviscera-Bioscience assays showed acceptable values (13% and 12% CV, respectively), although measured inter-assay variations were slightly higher than those declared by the manufacturers. R&D System-Quantikine^®^ showed the poorest performance in terms of inter-assay variation (20%). Kit descriptions provided by producers indicated a factor 10 difference in assay sensitivity between kits, ranging from 2 pg/ml to 20 pg/ml (mean 9.07 pg/ml) and even greater differences in the range of detection, from the narrowest range of 7.8–500 pg/ml for the Biosensis, Millipore-ChemiKine^TM^ and Promega-Emax^®^ kits, to the broadest range of 12–50,000 pg/ml provided by the Millipore-Milliplex^®^ assay, which uses the Luminex^®^/xMAP^®^ technology. All kits examined are able to detect BDNF within a restricted concentration range (from 7.8 pg/ml to 4 ng/ml), with the exception of multiplex assay which had a broader range (12 pg/ml to 50 ng/ml). Most studies including ours, report a range of BDNF concentrations between 8–46 ng/ml, with an average around 18–26 ng/ml for healthy Caucasian adults, and generally not beyond a minimum of 3 and a maximum 80 ng/ml when the extreme values are considered^32^,^35^,^43^. Therefore, a dilution step must be applied to process the samples, which may introduce a potential source of additional errors. Nevertheless, the results from our study suggest that all kits are able to detect serum BDNF reasonably well within the range of concentrations normally found in a healthy Caucasian population.

The sandwich ELISA assays tested here differed also for the type of capture and detection antibodies. In addition, capture antibody are generally pre-coated, with the exception of the Promega-Emax^®^ kit, which requires preparation by the experimenter. The Millipore-Milliplex^®^ kit exploits the Luminex^®^/xMAP^®^ technology, where magnetic microspheres, filled with a dye mixture, are coated with the capture antibody; this technology allows multiplexing and high throughput screening, but requires special equipment. Remarkably, the Biosensis kit is built on a unique strategy, because it uses the same antibody for both capture and detection, based on the fact that BDNF is naturally occurring as a dimer and therefore, once a monomer is captured, the other monomer is available for detection. The Biosensis kit provides also the quickest procedure (results in less than four hours for a full 96 well plate) while the kit by Promega-Emax^®^, which is probably the most used one, is time consuming (23–25 hours) because it requires an overnight plate coating, another potential source of variability. Of note, the Promega-Emax^®^ kit resulted to be the only assay among those tested, for which the species-specificity was not declared while the other kits were declared to be specific for detection of human BDNF. In addition, Biosensis and Millipore-ChemiKine^TM^ can also cross-react with BDNF from rodent species. Concerning the specificity for the different protein forms of BDNF, four kits recognized both pro- and mature BDNF forms while Aviscera-Bioscience and R&D System-Quantikine^®^ kits showed a remarkable preferential specificity for the mature form of BDNF, as indicated by the manufacturers. The ability of a diagnostic assay to distinguish between BDNF precursor and BDNF mature forms might represent a critical issue in specific clinical applications. Indeed, we recently proposed that altered biosynthesis of the different BDNF proteolytic forms may represent a common hallmark of neurological diseases with cognitive dysfunctions^8^,^10^. This hypothesis is based on studies in animal models and post-mortem human brains, which highlighted that different forms of learning and memory require either the pro-BDNF precursor or the mature BDNF form. In particular, decreased synthesis of mature BDNF, especially in hippocampus and prefrontal cortex, is associated with memory loss and learning impairment; while decreased production of pro-BDNF in amygdala is associated with dysfunctions in emotional control and finally, alterations of both mature BDNF and pro-BDNF can affect several cognitive functions at the same time^10^,^44^. Of note, specific kits for detection of pro and mature BDNF forms are now becoming available^43^,^45^. It remains to be determined if the ELISA kits specific for pro-BDNF have sufficient sensitivity to be appropriate for clinical applications.

Besides the technical issues discussed here, several studies investigated socio-demographic determinants and other factors that may affect serum levels of BDNF such as gender, age, Body Mass Index (BMI), urbanicity, smoking status, and alcohol intake. While there are conflicting studies regarding BMI^23^,^36^,^46^,^47^,^48^, concerning the effect of gender, our findings are in agreement with studies showing no gender difference for BDNF serum levels^23^,^36^,^47^,^49^. However, it was also reported that BDNF in whole blood or plasma was higher in women compared to men^30^,^32^ and several studies have suggested an interaction, with respect to circulating BDNF, between gender and age^23^,^26^,^36^,^47^,^50^. Serum BDNF resulted to be increased in association with nicotine assumption^51^,^52^ and to be decreased in association with high alcohol intake (>2U/day), but not with moderate alcohol consumption^23^,^48^. A final note regards the putative effects of the Val66Met polymorphism in *BDNF* gene on the levels of serum BDNF. The large majority of studies reported no association between Val66Met and serum BDNF in Asiatic, European and American populations^51^,^52^,^53^,^54^,^55^ as also summarized in a recent meta-analysis^56^. It is worth mentioning however, that multiple regression analysis highlighted that the Val66Met polymorphism may affect serum levels of BDNF through a complex interaction with gender and in response to specific treatments such as IFN-α^48^,^57^ or linked to neurophysiologic response associated with working memory processes^58^. These confounding factors are all to be taken in consideration in future studies aimed at defining a cut-off in serum BDNF concentration useful for diagnostic purposes.

Our protocol for serum preparation, stems from the comparison of a large body of available literature which converge on a few common points, such as blood withdrawal in the morning under fasting conditions, short clotting times and storage at −80 °C^23^,^24^,^27^,^28^,^29^,^30^,^31^,^32^. Nevertheless, a limitation of this study is that we could not validate a standardized method. Accordingly, a multi-laboratory effort is needed to define a standardized procedure for serum preparation for future clinical applications.

In conclusion, the availability of reliable blood-based biomarkers for diagnosis and therapy monitoring still remains a major unmet medical need in neurology and neuropsychiatry^1^,^59^. There is a general consensus that BDNF may represent an important measurable biomarker however, the poor reproducibility of BDNF measures has to date prevented its validation for clinical purposes. In this study, we identified Biosensis for total BDNF and Aviscera-Bioscience for mature BDNF as the kits which provide the most reproducible measurements of serum BDNF out of six commercially available kits. Nevertheless, future studies are needed to validate such method involving different laboratories and providing longitudinal analysis thus yielding the basis to obtain an accurate BDNF measure in human serum, suitable for future clinical applications.

## Methods

### Participants and sample collection

Forty healthy subjects (mean age 54 ± 6, range: 41–70 years old; 18/22 Females/Males ratio) participated in this study as normal controls. Subjects were enrolled at the Department of Transfusion Medicine of the Trieste University Hospital and signed written informed consent to participate to the study in accordance with the recommendations of the declaration of Helsinki and the Italian law DL n° 675 of the 31-12-1996. The experimental protocols were approved by the University Hospital’s Ethics Committee (Approval protocol n. 78/2010) and methods were carried out in accordance with the approved guidelines. Blood samples from healthy control subjects were collected between 9:00 to12:30, in fasting condition and let them clot for 1 hour at room temperature, followed by 1 hour at 4 °C. Serum was then separated by centrifugation at 2000 g for 10 minutes at 4 °C, aliquoted and stored at −80 °C until use in 0.2 ml tubes strips (Sarstedt, Multiply^®^ μStripPro).

### BDNF measurement

Serum levels of total BDNF were measured by using six different ELISA kits, as listed in Table 2: human BDNF ELISA Kit (Cat #: SK00752-01, Aviscera-Bioscience, Santa Clara, CA, USA), BDNF *Rapid*^TM^ ELISA Kit: Human, Mouse, Rat (2 Plates; Cat #: BEK-2211-2P, Biosensis Pty Ltd., SA, Australia), ChemKine^TM^ BDNF Sandwich ELISA (Cat #: CYT306) and Milliplex^®^ Map Human Pituitary Magnetic Bead Panel 2 - Endocrine Multiplex Assay, based on Luminex^®^/xMAP^®^ technology (Cat #: HPTP2MAG-66K, both from EMD Millipore Corporation, Billerica, MA, USA), BDNF Emax^®^ Immuno-Assay System (Cat #: G7610, Promega Corporation, Madison, WI, USA), Quantikine^®^ human BDNF Immunoassay (Cat #: DBD00, R&D Systems, Inc., Minneapolis, MN, USA). All samples were assayed in duplicate on each plate, in order to test also the intra-assay variation. A number of two plates per kit, of the same lot, were used over two different days, within the same week, in order to assess the inter-assay variation. The third replica for Aviscera-Bioscience, Biosensis and Millipore-ChemKine^TM^ kits was carried out at 1 year distance from the second and therefore, was performed using a different lot. Protocols were performed according to the manufacturer’s instructions. The optical density of each well was measured using an automated microplate reader (GloMax^®^-Multi Microplate Reader, Promega) or the Bio-Plex^®^-200 instrument (Bio-Rad) for the multiplexing assay measurements.

### Statistical analysis

For each kit, sensitivity and range were estimated based on the standard curves. Sensitivity was stated as equal to the value declared in the kit if the absorbance of the most diluted standard was at least 10% above the absorbance of the background (blank). Similarly, the range was confirmed equal to the declared one if the most concentrated standard did not produce an R2 value (linearity of the curve) under 90%. Because BDNF values were not normally distributed, data are presented as median, 25^th^–75^th^ percentile and range (see Table 2). To evaluate intra-assay variation, the BDNF values from the two different well, for each subject, were mediated and the coefficient of variation (CV) was calculated for all samples. Again, due to the non-normal distribution of the CVs, the median value was considered. Similarly, to assess inter-assay variation, the BDNF concentrations, for each subject, were mediated between the two or three plates tested and the median CV values were considered. Differences in the BDNF concentrations, as a repeated measure from two or three independent plates, were assessed using one-way ANOVA for repeated measures. Values of p < 0.05 were considered statistically significant. All statistical analysis and graphs were performed using SigmaPlot 11.0 (Systat Sofware, Inc.).

### Dot blot

Commercially available human recombinant pro-BDNF (Alomone Labs) and two types of mature-BDNF (Sigma and Alomone Labs) were spotted on a nitrocellulose membrane at the concentration of 10 pg and 1000 pg per lane, respectively. Together, were spotted BSA (1000 pg), as negative control, and the BDNF standards (between 25 pg and 100 pg, depending on the stock concentration) provided by the ELISA kits. Dot-blots were performed according to Abcam guidelines with minor modifications, and all steps were carried out at room temperature. Briefly, once proteins have been spotted, the membrane strips were dried and then non-specific sites were blocked by soaking in 5% BSA in TBS-T (20 mM Tris-HCl, 150 mM NaCl, 0.05% Tween20) for 30 minutes. ELISA’s primary antibodies were used at the concentrations suggested by each kit manufacturer in the diluents provided, when possible; Millipore (ChemiKine^TM^) and Promega-Emax^®^ antibodies were dissolved in BSA/TBS-T (0.1% of BSA in TBS-T). Monoclonal antibody anti-BDNF (Sigma) was used as control at a dilution from stock to 1:1000 in 0.1% BSA in TBS-T. All incubations with primary antibodies were performed for 1 hour and then strips were washed three times in TBS-T for five minutes each. As secondary detector, streptavidine-HRP was used (Pierce Biotechnology; 1:2000 in BSA/TBS-T), except for Promega-Emax^®^ polyclonal antibody (the anti-IgY-HRP provided with the kit was used) and for Promega-Emax^®^ and anti-BDNF (Sigma) monoclonal antibodies (the anti-mouse-HRP from Sigma was used, diluted 1:10000 in BSA/TBS-T). R&D System-Quantikine^®^ antibody is already HRP-conjugated and did not require further detection by a secondary antibody. Incubations were performed for 30 minutes, followed by three wash steps with TBS-T and then rinsed with TBS. As reaction substrate, ECL-prime (Amersham) was used and X-ray film exposed in the dark room to display the signals.

## Additional Information

**How to cite this article**: Polacchini, A. *et al.* A method for reproducible measurements of serum BDNF: comparison of the performance of six commercial assays. *Sci. Rep.* **5**, 17989; doi: 10.1038/srep17989 (2015).

## Figures and Tables

**Table 1:** BDNF ELISA Kits.

Company	Aviscera-Bioscience	Biosensis	Millipore	Millipore	Promega	R&D System
KIT Name	Human BDNF ELISA	BDNF *Rapid*^TM^ ELISA	ChemiKine^TM^	Milliplex®	BDNF Emax^®^ Immuno-Assay System	Quantikine®
Principle of the assay	Sandwich ELISA	Sandwich ELISA	Sandwich ELISA	Luminex®/xMAP® technology	Sandwich ELISA	Sandwich ELISA
Sensitivity (pg/ml)	5–8	2	7.8	2.5	15.6	20
Range of detection (pg/ml)	23–1500	7.8–500	7.8–500	12–50000	7.8–500	62.5–4000
BDNF standard	Human recombinant	Human recombinant	Human recombinant	Mix of BDNF 12500 pg +Prolactin	BDNF standard (type not declared)	Human recombinant
Coating/capture Antibody	Pre-coated α-BDNF antibody (not type declared)	Pre-coated α-BDNF antibody (mouse monoclonal)	Pre-coated α-BDNF antibody (mouse monoclonal)	Mix of: magnetic beads coated with α-BDNF Ab OR α-Prolactin(types not declared)	Manually coating with α-BDNF Ab (mouse monoclonal)	Pre-coated α-BDNF antibody (mouse monoclonal)
Primary detection antibody	Biotinilated α-BDNF antibody (type not declared)	Biotinilated α-BDNF antibody (mouse monoclonal)	Biotinilated α-BDNF antibody (mouse monoclonal)	Mix of: Biotinilated α-BDNF antibody AND α-Prolactin (type not declared)	α-BDNF Ab (chicken polyclonal)	α-BDNF antibody (mouse monoclonal)-HRP conjugated
Type of secondary detection	Streptavidin-HRP conjugate	Streptavidin-HRP conjugate	Streptavidin-HRP conjugate	Streptavidin-Phycoerythrin conjugate	Anitibody α-IgY-HRP conjugated	/
Sample dilution suggested	1:40 - 1:80	1:50 - 1:200	1:2 – to user optimization	1:10	1:4 – to user optimization	1:20 at least
Declared species cross-reactivity	Only human	Human, mouse, rat and others	Human and rat	Only human	Not specified	Only human
Processing time	6–7 hours	3–4 hours	21–22 hours	19–20 hours	23–24 hours	4–5 hours

Description and main characteristics of the tested BDNF ELISA kits, as declared by the manufacturers.

**Table 2:** BDNF ELISA kits performance.

Number of plates tested: 2	Aviscera-Bioscience (n = 38)	Biosensis (n = 39)	Millipore ChemiKine^TM^(n = 39)	Millipore Milliplex® (n = 38)	Promega Emax^®^ (n = 40)	R&D System Quantikine^®^ (n = 40)
Range ng/ml: min-max	14.1–32.4	13.6–41.4	14.5–58.5	8.7–32.4	12.1–27.5	15.4–46.2
Median ng/ml (25%–75%)	20.7 (18.5–24.4)	25.4 (21.5–32.0)	23.0 (18.6–25.9)	18.2 (14.2–22.2)	19.0 (16.9–21.7)	25.5 (21.0–30.5)
Declared intra-assay CV	4–6%	2–6%	4%	< 10%	2–9%	4–6%
Tested intra-assay CV	2%–3%	1%–1%	2%–3%	13%–14%	3%–7%	5%–6%
Declared inter-assay CV	8–10%	5–8%	9%	<10%	<9%	8–11%
Tested inter-assay CV	12%	5%	13%	18%	16%	20%
Tested processing time	**7** hour**s**	**4** hour**s**	**22** hour**s**	**20** hour**s**	**24** hour**s**	**5** hour**s**

Comparison between declared performances and actual tested performances of the specified BDNF ELISA kits. Median and range values, intra- and inter-assay coefficients of variation (CV) were assessed by measuring BDNF serum level from healthy volunteers (n = 38–40). All the kits, except Milliplex^®^, which is based on multiplex technology (Luminex^®^/xMAP^®^), are based on classical sandwich ELISA.
